# ‘Embarrassing the State’: The ‘Ordinary’ Prisoner Rights Movement in Ireland, 1972–6

**DOI:** 10.1177/0022009419863846

**Published:** 2019-08-28

**Authors:** Oisín Wall

**Affiliations:** University College Dublin, Ireland

**Keywords:** Ireland, prison reform, prisoners’ rights, protest, psychiatry

## Abstract

This article explores the early years of the campaign for ‘ordinary’, not politically-aligned, prisoners’ rights in Ireland. It argues that this campaign has often been overshadowed by the activities of ‘political prisoners’, who only constituted a small minority of prisoners in the period. The article follows the development and changing tactics of the ordinary prisoners’ movement, through the rise and fall of the Prisoners’ Union (PU) (1972–3) and into the early years of the Prisoners’ Rights Organisation (PRO) (1973–6), which would become the longest-lasting and most vocal penal reform organisation in Ireland, until the formation of the Irish Penal Reform Trust in 1994. It argues that the movement constantly adapted its tactics to address emerging issues and opportunities. Ultimately, it contends that by 1976 the PRO was an increasingly legitimate voice in Ireland’s public discourse on prisons. It shows that, although the campaign did not achieve any major penal reforms in this period, it had a significant impact on public debates about prisons, prisoners’ mental health, the failures of the penal system, and prisoners’ entitlement to human rights.

In September 1975 the front page of *The Irish Press*, a major national newspaper, carried a story about a 22-year-old prisoner, Karl Crawley, who had been taken to hospital for emergency surgery. The hospital’s spokesman stated that the man was recovering well, while the Department of Justice and prison authorities refused to comment. The Prisoner Rights Organisation (PRO) claimed that prison officers had not believed the man when he told them he swallowed eight sharp objects, and instead sent him to the Central Mental Hospital (CMH), the primary site of psychiatric care for most prisoners in Ireland in the early-1970s. He spent five days there before the need for surgery became apparent.^1^

The transfer of prisoners to both psychiatric and general hospitals was routine, Crawley had frequently been a patient of the CMH in the past, self-harm had long been endemic in prisons, swallowing dangerous objects was not uncommon, and there had been no references to this practice in the press in the five years before 1975.^2^ Given the everyday nature of this incident it is unlikely that such a story would have received any public attention three years earlier, let alone made the front page. However, this article argues that since 1972 a wave of activism had brought prisons into the public eye and created a media landscape that was more sympathetic to prisoners’ experiences and concerns.

The article foregrounds the campaign for ‘ordinary’ (i.e. not politically aligned) prisoners’ rights, which, it argues, has often been overshadowed by the activities of political prisoners in this period. The article follows the development and changing tactics of the movement, through the rise and fall of the Prisoners’ Union (PU) and into the early years of the PRO. In doing so it argues that, the movement constantly adapted its tactics to address emerging issues and opportunities and shows that, although the campaign did not achieve any major penal reforms in this period, it had a significant impact on public debates about prisons, bringing prisoners’ mental health, the failures of the penal system, and prisoners’ entitlement to human rights.

This article will focus specifically on prisoner rights groups in the Republic of Ireland. Throughout the 1970s the prison systems of Republic and Northern Ireland were subject to different legal structures and shaped by different policy considerations. Not least of these was the greater impact that ‘the Troubles’ had on the Northern Irish Prison Service. At times two out of every three prison beds in the North were occupied by political prisoners; in the Republic that number stayed below one in six. Secondly the distribution of politicals throughout the Northern prison system contrasts sharply with the republic’s concentration, from 1973, of politicals in Portlaoise and the Curragh Military Detention Barracks. Thirdly, while Ireland’s prison population remained largely stable for 10 years after 1972, in the North it grew rapidly until 1978. Finally, although a well-organised prisoners’ rights movement developed among ‘ordinary’ prisoners in the Republic, there seems to have been no equivalent movement in Northern Ireland, where prisoner activism was shaped by republican or unionist political affiliations.

To construct this history, I examined the PRO’s *Jail Journal*, the Department of Justice’s Annual Reports on Prisons, government reports and memoranda, and reports by diverse non-governmental bodies like the Prison Study Group at University College Dublin (UCD) which produced a report on the prison estate in 1972, the MacBride Commission which recommended largescale prison reform in 1979, and the Catholic Bishops’ Council for Social Welfare which conducted an extremely critical inquiry into prison conditions in 1982.^3^ However, these offer a very incomplete picture. At the heart of this article are more than 200 articles published in national and regional newspapers, including the* Irish Press, Irish Independent*, *Evening Herald*, *Cork Examiner*, and *The Nationalist and Leinster Times*, between 1972 and 1976. This article not only constructs a thorough timeline of the movement, but also attempts to assess the relative impact of the events on public debates.

The formation of the first Prisoners’ Committee in 1972 is a natural starting point for this article because the Committee is the first significant piece of self-organisation by ‘ordinary’ prisoners in Ireland. Although this group spawned a long-lasting movement, this article focuses on the period before 1976. This was the movement’s most frenetic period, during which the strategy centred on the street-level organisation. After 1976 the strategy shifted to research and policy-focused tactics, this can be clearly seen in the almost complete disappearance of the individual narratives that had been a staple of the first three years of the PRO’s regular magazine, *Jail Journal* (1973–6), in favour of more objective articles examining budgets and visiting committee reports.^4^ While the PRO never became an ‘elite’ ‘approved group’, as Mick Ryan described The Howard League in the UK, the sociological shift did lead it away from its prisoner-led origins.^5^ This shift was one of the causes of a split in the PRO in July 1977, when five key organisers, who felt the PRO had become overly academic, politically influenced, and ‘divorced from the day-to-day reality of running a prisoners’ association’, left to form the short-lived Prisoners Committee.^6^

As William Murphy notes, prison has a special place in Irish culture; all of the members of the first Executive Council of the Irish Free State had been imprisoned, and men who died on hunger strike a century ago, like Terence MacSwiney, remain household names for many people.^7^ More recently, in the 1980s, there was widespread support for Northern Irish hunger strikers and the ‘Free Nicky Kelly’ campaign.^8^ Indeed, to see the continued cultural significance of imprisonment one need only look at the lengthy queues, waiting to see the cells of the 1916 rebels, outside Kilmainham Gaol Museum, one of the most popular tourist attractions in Ireland. The literature on Ireland’s prisoners’ protests in the twentieth century has been shaped by this tradition and is focused on activities of political prisoners.^9^ The ‘ordinary’ prisoner rights movement has been touched on in very few histories of the Irish and international penal systems. For instance in Ian Miller’s work on force-feeding, Shane Kilcommins, Ian O’Donnell, Eoin O’Sullivan, and Barry Vaughan’s survey of crime and punishment in Ireland, and Cormac Behan’s work on prisoners’ enfranchisement, as well as in broader histories like Diarmaid Ferriter’s *Ambiguous Republic*, the topic is touched on only briefly and then usually to illustrate a broader point about the tensions in prisons in the 1970s or as background to a gradual shift towards a rehabilitative policy.^10^ Mary Rogan’s work goes into more depth when she argues that the campaign did not affect institutional change but rather instilled ‘suspicion, fear and hostility’ in policy makers.^11^ While this is true, Rogan’s focus on policy makers does not account for the pressure the movement exerted through public opinion, which could be seen as a key element in the commissioning of the first official inquiry into the prison system, the Whitaker Report, in 1983 and which certainly influenced its findings.^12^

In 2018 Cormac Behan compared the state’s reactions to the campaigns of the PU and those of ‘politically-aligned’ prisoners. He argued convincingly that the Provisional IRA’s concerns were accommodated informally making the imprisoned IRA de facto ‘political prisoners’ while allowing the state to avoid formally acknowledging that status. Meanwhile, the ‘ordinary’ prisoners’ demands were ignored, and their organisation was suppressed.^13^ The suppression of prisoners’ organisations is something that we will see borne out throughout this article, particularly in the suppression of the PU and governments’ attempts to delegitimise the PRO.^14^

As with other civil rights movements in 1970s Ireland, women played an important role in the prisoner rights movement. Maura Bates was a founding member of the Ad Hoc Committee for Prison Reform, and the PRO was inextricably linked with activists like Máirín de Burca and Margaret Gaj, who were also prominent in the Irish Women’s Liberation Movement. However, there are very few references to female prisoners in this article. This is primarily because of the small numbers of women in Irish prisons. In this period women never constituted more than three percent of the average prison population.^15^ Moreover, this population was split between Mountjoy and Limerick Prisons, making it easier to control and harder to mobilise. The women’s sections of the prisons on their busiest days in 1972 never housed more than 25 and 18 prisoners respectively and their average occupancy was only 13 and nine. Neither section was ever big enough to mount a logistical challenge to the prisons’ authorities.^16^ There were also restrictions on women’s communication with the outside world. In its second issue, the PRO’s *Jail Journal* claimed that female prisoners were not allowed pencils or pens, supposing that ‘it must be to prevent them smuggling out incriminating notes’.^17^ If that was the intention, it was effective. The early years of the Prisoner Rights Organisation were fuelled by information in secret letters from inside Mountjoy and Portlaoise that were almost exclusively from men.^18^

By the early-1970s the Irish prison system was riven with challenges and contradictions. It had been largely neglected since the foundation of the state and was struggling to keep pace with rapid social change. The average daily prison population had remained under 700 for four decades before 1968. Many prisons were closed and the adult population was consolidated in Limerick Prison, Mountjoy Prison (Dublin), and the high security prison in Portlaoise.^19^ In the early 1960s Charlie Haughey, then Minister for Justice (1961–4), tried to modernise the prison system and instituted a number of reforms: expanding the parole service, developing the first psychiatric service in Mountjoy, creating a hostel for pre-release prisoners, and expanding educational and work release programmes.^20^ When Haughey left the Department in 1964, the political drive behind these reforms gradually petered out. In 1973 a coalition, led by the more conservative Fine Gael party, came to power and Patrick Cooney became the Minister for Justice (1973–7).^21^ Cooney adopted a decidedly less progressive stance; telling TDs that ‘no institution and no system can rehabilitate anybody […] rehabilitation is and cannot be the only consideration’.^22^

Another change was that from 1958 the average daily prison population began to rise gradually. This became more extreme in the late-1960s, rising almost 70 percent from 615 (1968) to 1,035 (1972).^23^ This corresponded to a growing crime rate that saw the number of indictable crimes reported to the Gardaí Síochána (police) rise above 20,000 for the first time in 1968, pass 30,000 in 1970, and almost reach 40,000 by 1972.^24^ This was also a period of demographic change in prisons. Between 1967 and 1977 the proportion of people sentenced to prison for longer than 12 months rose consistently. In 1967 only one in every 16.5 people sentenced to prison was sent down for over a year; by 1972 this number had become one in 8.5, and by 1977 it was one in every 4.2. We may even speculate that the combination of prisoners having more time to develop social bonds and their prolonged subjection to the deteriorating prison conditions, may have created the conditions in which the PU became possible.

There was also limited opportunity for prisoners to officially protest their conditions. Prisoners could complain to the governor or to their prison’s Visiting Committee, a group that was supposed to provide civilian oversight to the prison. However, both governors and committees operated at the pleasure of the Department of Justice and were responsible for meting out punishments and maintaining discipline. As a result, prisoners often distrusted them, indeed the Portlaoise PU described their Visiting Committee as ‘biased and hypocritical’ and demanded that it be ‘immediately dissolved’.^25^

Throughout this period educational and medical facilities were, at best, basic. Since 1947 every prison was required to provide classes in reading, writing, and arithmetic for four hours a week to eligible prisoners.^26^ By 1972, however, neither Mountjoy nor Portlaoise had trained teachers and basic literacy classes were provided by uniformed officers; in Limerick these classes were provided on a voluntary basis by a retired nun. What is more, these meagre facilities were only available to a small number of prisoners who could not read or write. In Mountjoy, for instance, only 13 of the average population of 416 attended classes regularly.^27^ Work programmes and training schemes were also limited, in spite of Haughey’s reforms. For instance, in September 1973 in Portlaoise over 60 percent of working prisoners were employed in unskilled labour on the prison farm and gardens, cutting turf, or on cleaning duties with only six percent involved in trades.^28^

The psychiatric provisions were also basic across most of the prison system, in spite of evidence that up to 50 percent of prisoners needed some form of psychiatric care.^29^ Mountjoy had the best provision for psychiatric care: in 1972 the full-time Medical Officer in Mountjoy was a trained psychiatrist, and there was also a visiting psychiatrist who came to the prison once a week. His main role was to provide prescriptions, but not social, occupational, or talking therapies.^30^ Neither Limerick nor Portlaoise had any psychiatric staff at all and prisoners’ crises were either dealt with as disciplinary matters or by transfer to the CMH.^31^ The CMH was the main provider of intensive psychiatric care to prisoners. In 1972, 70 of the hospital’s 120 patients were ‘justice patients’.^32^ Although prisoners were meant to be committed to the hospital by a psychiatrist, in practice, many prisoners saw a psychiatrist for the first time after they had been transferred to the hospital. These committals were brief and ‘justice patients’ were returned to prison as quickly as possible. Even as the main psychiatric service provider to prisoners, a study in the early 1970s noted, ‘the psychiatric treatment in Dundrum [the CMH] is very limited. There are no facilities for rehabilitation.’^33^ While limited prison psychiatric services were not unusual internationally, Ireland lagged behind several of its European neighbours. England, for instance, began the process of integrating the Prison Medical Service (PMS) and the National Health Service (NHS) in the mid-1960s leading to the appointment of seven consultant forensic psychiatrists by 1975. The British system was far from a model of best-practice and there were many critical flaws in the system. Nicholas Duvall cites a psychotherapist at Wormwood Scrubs who claimed that to ‘suggest that the NHS is at this time at least capable of taking over the Prison Medical Service is to lose contact with reality’ and while he may have been correct, the very presence of a psychotherapist inside the prison system indicated the wider range of treatments than in Ireland.^34^

The conditions in Irish prisons were not very unusual by international standards, and nor were the protests. A year before the PU was formed in Portlaoise, prisoners in Attica, New York, seized control of the prison. The four-day Attica uprising was, in many ways, the culmination of years of protests, as well as radical education and organisation within the prison system and it brought the American prisoner rights movement to global attention.^35^ In Ireland the story was received with some sympathy: even conservative newspapers like the *Irish Independent* and the *Evening Herald* described it as a ‘rebellion’, rather than using the more pejorative terms like ‘riot’, and showed sympathy for the ‘brutal’ treatment of prisoner-activists after the uprising.^36^ In the months after the Attica revolt, prisoners in the United Kingdom began to organise various kinds of strikes and in spring 1972, a group called the Preservation of the Rights of Prisoners (PROP) emerged to coordinate these protests.^37^ In August, they led 10,000 prisoners in a 24-hour general strike and over the next few years they continued to organise and campaign for penal reform, and sent organisers abroad to meet with other activist groups, like the PRO.^38^ In France the Comité d’Action des Prisonniers (CAP) was also formed in 1972. It went on to organise prisoners’ protests across for the country for over a decade.^39^ The CAP’s work expanded on that of the Groupe d’Information sur les Prisons which had brought prisoners’ stories to the public as a way of raising awareness and challenging the secrecy of the prison system.^40^ Although the tactics resemble those of the PRO I have not yet found any direct lines of influence. There were also a series of prisoner riots and reprisals by prison officers in New South Wales, which became known as the ‘Bathurst batterings’.^41^ In 1973, the Prisoners’ Action Group (PAG) was formed to campaign for a public enquiry into, and the ultimate abolition of, the prison system. The PAG also brought individual prisoners’ stories to the courts and to the press.^42^ Although there are clear parallels here with the PRO’s activities in the same period I have, again, not found any direct links between the organisations.

While the 1960s had been marked by penal reform in Ireland, the 1970s were a decade of crisis management.^43^ The Department of Justice focused on managing new problems, like overcrowding and protests, as they emerged. For the most part this meant moving prisoners into the Curragh military detention barracks, opening new prisons, and expanding older prisons.^44^

On 13 November 1972, 80 prisoners in Portlaoise, almost half the prison’s population, staged a ‘silent protest’.^45^ After their evening tea, the prisoners sat down in the recreation area and refused to return to their cells. Garda reinforcements were called in, as was the army riot squad.^46^ A conference was held between the prison governor and representatives of the prisoners, The Prisoners’ Committee. The Committee presented the governor with a list of complaints about conditions of cells, the lack of recreational facilities, and the quality of the food. The news coverage primarily represented it as ‘unrest’ or a ‘disturbance’, rather than a coordinated protest and paid little attention to the prisoners’ complaints or demands until May 1973 when two former members of the PU, Tom Bourke and Simon O’Donnell, began publicising the reasons for the development of the PU through the newspapers and public meetings called by the Ad Hoc Committee on Prison Reform, the precursor to the PRO.^47^

At 11pm the prisoners returned to their cells. The next morning, they refused to work and the Gardaí and army were called out again. Another conference was held and the men returned to their cells. That evening the Portlaoise Visiting Committee arrived at the prison where they placed 79 prisoners on a punishment diet.^48^ Although this was supposed to be reviewed after 28 days one prisoner recalled it continuing for 57 days, earning the committee the moniker of ‘the bread and water committee’. ^49^ There followed widespread allegations of brutal reprisals by prison officers. A rumour circulated that several prisoners had gone on hunger strike while others had self-harmed, which was laughed-off by a member of the Visiting Committee who told a reporter: ‘a couple of prisoners had scratched arms’.^50^ This dismissive attitude was not uncommon. The protest itself received next to no media coverage. One of the few contemporary articles about the aftermath of the protest, tucked away on page 19 of the local *The Nationalist and Leinster Times*, used the light-hearted title ‘Prisoners on diet’ referring to the prisoners’ punishment.^51^ Indeed, this is not surprising, hunger strikes and other forms of self-harm were common forms of protest in prisons around the world. Moreover, it has a particular historical weight in Ireland. William Murphy describes some of the debates over the tactic, by the suffragettes, trade unionists during the 1913 Lock Out, and by Irish Republican prisoners.^52^ However, it was not only common among politically motivated prisoners, in England between 1913 and 1940, there were over 1000 incidents of hunger strikes in prisons, at least 80 percent of which were by ‘ordinary’ prisoners.^53^ Although coherent records of hunger strikers were not kept after 1940, Ian Miller has argued that hunger striking continued at a similar rate throughout most of the century.^54^ Others have reported hunger striking and self-harm as common methods of protest throughout the period in countries as disparate as South Africa and France.^55^ Indeed, today there is an established medico-legal and sociological literature on institutionally powerless prisoners use of self-harm and hunger strikes as a way or exercising autonomy over their own bodies and an indirect power over others.^56^

The protests by the prisoners in Portlaoise were taken more seriously in January 1973, when they coincided with extensive Provisional IRA protests. For instance, in early-January the *Irish Independent* reported that some of the Portlaoise prisoners’ demands had been met: ‘They were given new beds, tooth brushes, toothpaste and new hand basins’ but that they were still angry.^57^ Almost as soon as the punishment diet was over, they came together again. On Sunday 28 January 1973 prisoners formed the PU, and elected a committee including two secretaries and shop stewards.^58^ According to one activist, the prison authorities intimidated members, forcing the president and five members to resign. In spite of this the PU had grown to 93 members by the end of March. The union had several false starts. In early-February it smuggled out a letter warning of, or perhaps threatening, an impending riot.^59^ In late-February it called for a hunger strike unless facilities were improved and the victimisation of PU members ended.^60^ There is no evidence that either of these threats materialised. In early-March a group of 15 civilians staged a demonstration in Portlaoise town in support of the PU. The protest was less than successful and was mocked in the press for picketing the shop of one Mr Clear with placards reading ‘Clear Out Clear’, under the mistaken impression that Mr Clear was on the Visiting Committee.^61^

The Minister for Justice, Patrick Cooney, simultaneously denied the existence of the PU and claimed that it had been established by ‘subversives’, the euphemism for the wide range of physical-force republicans.^62^ There is little evidence to support this implied conspiracy. While the political prisoners waged an extensive, and well publicised, protest campaign inside prison, their demands were rarely for reforms that would affect ordinary prisoners, instead they had long seen prison as another ‘theatre of revolutionary war’.^63^ The Provisional IRA held itself separate from ordinary prisoners, insisting they occupy different landings in prisons until 1973, when they were all moved to Portlaoise and the Curragh Military Detention Barracks. However, there were some similarities and connections between the PU and the politicals. The PU deployed similar tactics to those that had been used by political prisoners and suffragettes, including labour strikes, sit-ins, and hunger strikes.^64^ It would be easy to overstate their tactical similarities, given the unbalanced power-relationship between prisoners and the institutions and the limitations it puts on prisoners’ tactical options. In their early months, they also smuggled out communiques through Saoirse, an organisation campaigning for the release of Official IRA prisoners which controlled one of the few unofficial lines of communication.^65^ There may also have been deeper connections than this to the left-wing of the republican movement. In 1974 a memorandum, prepared by the Department of Justice to make the case for continued segregation of prisoners in the Curragh, claimed that prisoners from the revolutionary socialist and republican organisation Saor Éire had originally fomented the PU’s campaign.^66^ However, the PU always disputed these accusations. In May 1973 an early PU secretary, Simon O’ Donnell, emphasized that the union was not politically affiliated, claiming that of the first 93 members only four had political affiliations, O’Donnell himself and another man were members of the Maoist, anti-nationalist, British and Irish Communist Organisation and two men ‘had sympathies with’ Official Sinn Féin.^67^ He also points out that there were tensions between the PU and the politicals, such when the leaders of the PU were eventually moved to the Curragh their comrades were concerned that they would be attacked by political prisoners.^68^ In spite of these denials, however, the accusation that the PU, and later the PRO, was under the influence of the republican movement persisted, not least because of the vocal involvement of Maurín de Burca who was a prominent activist in both Official Sinn Féin and the PRO. Throughout the 1970s the PRO continued to insist that it had no political affiliation, but the accusations caused tensions within the group. For instance, in 1974 the PRO publicly chastised de Burca for an unauthorised political statement she made on behalf of the organisation; the PRO’s official rebuke reiterated ‘the Prisoners’ Rights Organisation is a non-political organisation and does not involve itself in or comment on political matters’’^69^ Even the Prisoners’ Committee’s statement in 1977 announcing its split from the PRO praised the group for having resisted various attempts to make it an ‘adjunct’ of a political group, although they do note that an unspecified group had finally managed to co-opt the PRO.

The relationship between the prisoners’ movement and the Prison Officers’ Association (POA) was also fraught. In 1973 members of the PU brought a series of charges against prison officers alleging a variety of crimes including torture, which the POA referred to as a ‘smear campaign’. In response they started a legal aid fund to pay for the defence of any members against whom criminal charges were brought and met the Minister for Justice to discuss legal protection for their members. The POA’s spokesman told the press that the PU was ‘a mafia type organisation … a disreputable body completely lacking in ideals’.^70^ The Prisoners’ Rights Organisation, on the other hand, had a more complex relationship with prison officers. They stressed that they ‘have no fight with prison officers’ and at times reached out to the POA for discussion and cooperation, but there was also clear animosity.^71^ The tension between these two stances is best summed up in two consecutive issues of the *Jail Journal*. An article in 1976 described the terrible working conditions that prison officers endured, how they too came from working-class backgrounds, and how the PRO should offer them solidarity.^72^ In the next issue, however, the PRO’s distrust of officers and their perceived complicity in the abuses of the prison system is evident in a cartoon of an officer who, finding a man hanged in a cell, calls out ‘Chief! Another one with a heart attack!’.^73^

Initially the PU’s demands were practical and firmly focused on the needs of prisoners rather than broader structural reforms. They demanded union recognition, an extra hour’s exercise each day, and overcoats. The demands were not only rejected by the prison authorities, but the prisoners were punished for making them. Those who wanted overcoats were moved from working in ‘fairly warm sheds’ to ‘pulling carrots with bare hands in the icy mud’, others were threatened with a punishment diet unless they left the PU.^74^ In May, 1973, 70 prisoners were refused recreation until they left the PU, and the PU’s president was punished for speaking to a group of prisoners.^75^ In addition to these official punishments, members alleged regular punishment beatings from prison officers.^76^

After their first protests the PU’s demands became more structural and widespread. On 8 May it smuggled a list of 11 demands out to the newly-formed Ad Hoc Committee for Prison Reform which printed them in the *Jail Journal*.^77^ Again, they included recognition of the PU and day-to-day needs like an end to restrictions on tobacco and food, an end to mail censorship, and improved facilities for education and physical exercise. However, the list also demanded some structural reform of the prison system: including the reformulation of the Visiting Committees and Parole Boards to include social workers, sociologists, trade unionists, and representatives of the PU.^78^ While the press did not reproduce the full list, partial lists of the demands were regularly included in articles about the PU and, later, the PRO, and questions were also asked in the Dáil (parliament) which clearly alluded to the demands, even though they fell short of mentioning the organisation’s name.^79^

On the morning of 23 May (six months after the initial silent protest), the PU members staged another sit-in, demanding an immediate meeting between their representatives and the Minister for Justice. The governor called in Garda reinforcements to move the prisoners back into their cells. At midday, before the Gardaí had time to enter the prison, the prisoners voluntarily returned to their cells. This time the focus of the punishment was on the four ‘ringleaders’ who received dietary punishment and were denied recreation, while the other protesters only forfeited their weekly food parcel privilege.^80^ Unlike the previous actions, this protest attracted significant public attention, thanks to the publicity of the newly formed Ad Hoc Committee for Prison Reform who, acting as the PU’s voice on the outside, smuggled letters out of the prisons. The prisoners’ position was widely reported, as was the speech by Minister for Justice, Patrick Cooney, to the POA in which he claimed that the PU was being run by ‘a small group of violent long-term prisoners’ who were ‘not interested in “prisoners’ rights” or anybody else’s rights, but in projecting themselves into a Mafia-like position as power bosses within the prisons’.^81^

Although both the November and May protests were met with harsh punishment and informal retaliation from prison officers, Noel Lynch and Danny Redmond, President and Secretary of the Portlaoise PU, were able to claim limited victories. In a letter smuggled out on toilet paper, they announced that their protests had resulted in a new diet programme and the beginning of the construction of a new toilet block.^82^ This letter, the only one to be published in a national newspaper, was the PU’s first and last public declaration of success. Even this modest boast may have been too much of a challenge to the prison authorities. On 13 September, five days after the letter was published, prison officers came to remove four leaders, including Lynch and Redmond, to the Curragh Military Detention Barracks.^83^ Officially the transfer was to relieve overcrowding, but the timing and the selection of prisoners suggest other motives. The men barricaded themselves into their cells. The authorities instituted a bread and water diet for the leaders and a widespread sit-down protest began, during which 14 prisoners climbing onto the roof.^84^ Over the next two days prisoners took shifts sitting on the roof in groups of five, entertaining themselves by singing and shouting at passers-by.^85^ While this was happening two other protests broke out in Mountjoy. One was a hunger strike for political status by provisional IRA prisoners; the other was the first protest by the recently formed Mountjoy Prisoners Union. This new formation claimed to represent all the prisoners in Mountjoy (presumably excluding the politicals) and that they were protesting for ‘more humane conditions for prisoners and to highlight the lack of proper educational, medical and recreational facilities in the prisons’.^86^ It remains unclear whether there was any coordination between these three protests although the Portlaoise protests were widely reported, so the Mountjoy prisoners probably knew about them.

When the last prisoner climbed down from the roof of Portlaoise on 15 September the PU leaders were not immediately removed from Portlaoise, but they were punished for their ‘mutiny’ with a bread and water diet. The PU tried to publicise this a week later when Richard Power, one of the leaders, appeared in court in Waterford applying to have a wallet returned to him.^87^ The court appearance gave him the opportunity to publicly announce that he and two other PU leaders had been put on a punishment diet and that, in protest, they had undertaken a hunger and thirst strike. The judge, deciding that the application was vexatious, adjourned the proceedings ‘until you are on full food or dead’ and pre-judged the case: ‘I will strike it out then’.^88^ While the protests had established prisoners’ conditions as a subject for public debate, this did not mean that prisoners had a legitimate voice in that debate and the hardships of individual prisoners continued to fall on unsympathetic ears.

Later in September the leaders of the Portlaoise PU were quietly moved to the Mountjoy ‘B Base’, basement solitary confinement cells. On arrival the organisers, described in a Department of Justice memorandum as ‘extremely resourceful agitators’, made contact with other prisoners through their ventilation windows and within a month the Mountjoy PU attempted another protest, similar to that in Portlaoise.^89^ With the help of the PRO the prisoners smuggled out a letter on 6 October reiterating their demands for the abolition of dietary punishment and solitary confinement; extended visiting times, recreational time, remission, and a parole system; improved education, trade courses, medical and psychiatric care; and the recognition of the PU.^90^ After handing over the letter, 20 prisoners tried to climb onto the roof but were prevented from doing so by prison officers. The press’ tendency to distrust prisoners can again be seen in the *Irish Independent*’s report on the incident, which was called ‘Knives used in jail’ and reiterated the POA’s claim that officers had been attacked with ‘bars, bricks, trays, pots of hot tea, broken delph[sic], pieces of steel piping and knives’, injuring 12.^91^ The article did not mention the PRO’s claim that the prisoners had not used violence. Two weeks later the PU smuggled out another letter accusing prison officers of ‘severely beating’ 27 prisoners, 10 of whom had to hospitalised, and calling for an inquiry into the events. Later that month the PRO claimed that these 27 prisoners had also been put in solitary confinement.^92^ On 11 November 1973, the PRO released a statement that 25 leaders of PU had been moved to the Curragh military prison.^93^

This was not the end of protests inside prisons, or even protests by the members of the PU, but it was the end of the PU as a coherent organisation. When prisoners protested after this, they did not demand the recognition of a PU and the press no longer referred to the organisation.^94^ It appears that the harsh punishments and the threat of years of isolation in the Curragh, the only military prison in the West to hold civilian prisoners at the time, deterred further activism. However, the PU had brought ‘ordinary’ prisoners’ conditions to the public attention and started a long public discussion. A week after the leaders were removed, members of the press were given the first tour of the Irish prisons since 1960.^95^ This was largely a response to the PU’s campaign which had raised questions about systemic issues in the prisons. After the tour the papers reported on the poor conditions in prisons, but their critiques were tempered by their acceptance of the government’s narrative of improvement.^96^

In May 1973 the Ad Hoc Committee for Prison Reform was established. At the centre of the Committee was Tom Bourke, a 39-year-old Limerickman who had spent a third of his life in British and Irish prisons. In Portlaoise Prison, which he had left two weeks before the press conference, Bourke had been angered by the lack of education and training opportunities. In particular he had been refused permission to do an accountancy correspondence course, and instead spent over two years working in the tailors’ shop making prison uniforms, a skill that was not transferable to his life as a house-painter.^97^ Bourke had been involved in the PU since its formation and had been the union’s secretary. Alongside him, the committee included Conal Gibbons (a UCD law student), Patrick McCartan (a UCD law student and secretary of the National Civil Liberties League), John Kearns and Matt Merrigan (organisers with the Amalgamated Transport and General Workers Union), and Maura Bates (a solicitor).^98^ In later years Gibbons and McCartan both became circuit court judges; and Merrigan, who was already the national secretary of the ATGWU in 1972, became the President of the Irish Congress of Trade Unions.

It was only after the Committee started working on behalf of the PU that the union garnered widespread public attention. As well as publicising the PU’s communiques, the committee sought to organise a campaign of protests across the country and undertake research into the prison system. At their first press conference the Committee announced that they were ‘compiling a dossier for the Minister for Justice on the whole penal system’ and the next day they released a statement calling for a public inquiry into the handling of the recent protests by the PU. The Committee quickly grew and on 12 July they held a public meeting reforming themselves as the Prisoner Rights Organisation (PRO), which would become the longest-lasting and most vocal penal reform organisation in Ireland, until the formation of a new group called the Irish Penal Reform Trust in 1994.^99^

In the first issue of their magazine, *Jail Journal*, the PRO laid out their aims, which were quite clearly modelled on the PU’s 11 demands.^100^ However, they also represented an evolution within the movement. They still addressed the day-to-day concerns of prisoners, for example in their access to literacy classes, increased remission, and extended recreation time. However they also proposed ambitious systemic reforms like the destruction of criminal records after five years; the stamping of insurance cards in prison; and a coherent aftercare programme that would ensure that a released prisoner had adequate housing and employment. This growing ambition may have come from the mix of people involved in the PRO, ranging from former prisoners to civil rights activists, and it speaks to a broader concern with addressing the root causes of imprisonment and recidivism. The changing makeup of the group meant that they began to speak with what could be seen as a more ‘respectable’ voice. New activists came to prominence like Pat McCartan, who by the mid-1970s was a promising young solicitor; and Joe Costello, a teacher in a private girls’ school who went on to become a minister of state. The initial statement made by Tom Bourke had been treated with some scepticism in the press, for instance the *Nationalist and Leinster Times’* article about the press conference undercut the new group’s credibility with the clause ‘assuming Mr Burke [sic] to be telling the truth’. However, as the group grew their spokespeople tended to be more experienced campaigners often without direct experience of prison. Their use of the language of rights also indicates an attempt to shift the public discourse towards acknowledging the humanity of prisoners, something which they sought to reiterate wherever possible. The language of rights was particularly weighted in Ireland in the 1970s. It builds on a late-1960s discourse of civil rights, then an influential theme in international news, particularly relating to America. More locally, it became a common lens through which to understand social issues in Northern Ireland. References to prisoners’ rights also drew inevitably on the international cache of the prisoner rights movement in America and the British prisoners’ union, PROP, both of which were treated with sympathy, particularly after PROP protested discrimination against Irish people in British jails. Finally, the language of rights relied on human rights discourse, which was increasingly prominent in Ireland in the 1970s, particularly after the Republic of Ireland took the United Kingdom to the European court of Human Rights over the treatment of detainees in Northern Ireland.

In the early years the PRO focused on two main tactics: picketing and using individual prisoner’s stories to spark public debate. Just over two weeks after the organisation was formed, 50 protesters held a picket outside Mountjoy Prison calling for an end to the ‘mental and physical torture’ of the current penal system. According to one journalist, the organisation threatened that this was ‘the first salvo in a campaign which would become nation-wide within the next few weeks’.^101^ A month later 12 PRO activists picketed a dinner dance that the Minister for Justice was holding for International Police Association delegates. They called on the Minister to follow through on the ‘radical noises’ he had made while in opposition.^102^ In September, 40 activists picketed the Department of Justice, protesting poor psychiatric provision in prison.^103^ In October they picketed Portlaoise Prison protesting the general prison conditions.^104^ In spite of their early enthusiasm for a nationwide campaign of pickets, once the PU collapsed inside the prisons, the Organisation’s demonstrations became less frequent. Throughout 1973/4 they continued to periodically picket institutional targets like the Department of Justice and Mountjoy Prison to highlight events like the hunger strike of 11 former PU members in the Curragh. However, these protests had little public impact, usually limited to one or two short newspaper articles.^105^

In May 1975 they tried a different approach and picketed the home of Richard Crowe, the senior civil servant with responsibility for prisons.^106^ They called for an inquiry into prison conditions in light of the suicides of three men and the attempted self-immolation by a fourth. In light of the PRO’s plan to picket his house every Saturday, Crowe won a High Court injunction forbidding it.^107^ While the picket itself, like its previous demonstrations, received little attention, the subsequent court proceedings won the PRO widespread press coverage that brought the question of prison suicides further into the public consciousness and conversation.

This was quickly followed by a second legal battle. In July 1975, seven members of the PRO were arrested while picketing outside the trial of Karl Crawley, whose story opened this article. The protesters were arrested after a Garda found leaflets, pleading for Crawley to be transferred to the CMH, under the bibles on the jury’s benches.^108^ The judge believed this would prejudice the jury and the seven activists were sentenced to 12 months’ imprisonment. They appealed the case and, although not acquitted, were granted the benefit of the Parole Act and released.^109^ Again, like the action that led to the High Court injunction, the original picket received minimal press until legal proceedings made it national news.^110^ The conversation about Crawley’s case built on the discussions about suicide in prison and fuelled the media’s conversation about mental health in prison well into 1976.

Pickets and court cases were not the only tactic that the PRO used. Aware of the negative representation of prisoners in the press, they used individual prisoners’ experiences to draw attention to broader issues. The first issue of the *Jail Journal*, for instance, included articles about the experiences of five men, highlighting the treatment of juveniles, violence by prison officers, a High Court challenge to the labour rights of prisoners, the monotony of prison life, and the lack of mental healthcare. Until 1976 there were between four and seven individual’s stories in most issues, each highlighting endemic problems, from the lack of treatment for alcoholics to the institutional challenges faced by prisoners’ families. Each story gave voice to someone whose experiences represented something integral to the prison system, but which was usually obscured by the system’s secrecy.

These stories were not only a narrative device to communicate a policy, nor were they simply a way of humanising prisoners for the public. They also determined, to a certain extent, the direction of the PRO’s activities. For instance, when Joseph Kavanagh killed himself in Mountjoy in August 1973 it was the first prison suicide widely reported by the media in over 10 years, and it marked a significant change in the prisoner rights movement’s approach to mental health in prison.^111^ Previously the PU had been primarily concerned with the impact of the close supervision of family visits by prison officers, often suggesting that this had led to marital and mental breakdowns.^112^ However, telling the story of Kavanagh focused the PRO’s attention on the deteriorative effect of prison and the lack of psychiatric care offered to prisoners.

Kavanagh was a 38-year-old man from County Wicklow on remand in Mountjoy for assaulting his aunt. The night before his trial he used bedsheets to hang himself in his cell. In the five years before his arrest he had been a regular patient of Newcastle Psychiatric Hospital in Wicklow and, when he was arrested, both his brother and the Garda responsible for transporting him said he clearly needed psychiatric care, which he did not receive on arrival in Mountjoy.^113^ The PRO alleged that the precautions to prevent prisoners like Kavanagh from self-harming were ‘infantile’ and that ultimately the Minister for Justice was criminally negligent.^114^ The PRO’s response to Kavanagh’s death established a tactic that they would repeatedly use to great effect. They would publicise an individual’s suffering, using it as a way to highlight endemic problems within the prison system and to advance their case for prison reform. When Kavanagh’s death was announced the PRO released a statement declaring: ‘Many concerned people have told him [the Minister for Justice] bluntly about the kind of prison conditions for which he is ultimately responsible. His reply has been to attack those who would try to set up a prisoners’ union.^115^ Later, in October, the PRO won the right to be represented at the inquest into Kavanagh’s death, where they argued for a full public inquiry into the circumstances surrounding the death, and for the appointment of a full-time psychiatrist to Mountjoy. The coroner rejected both calls but did recommend that all newly committed prisoners should receive a medical and psychiatric assessment on arrival.^116^

The PRO used this tactic seven times between 1973 and 1976. Each time they managed to turn individual tragedies into major public debates about prison conditions. The most intense period occurred in 1975, when Kevin Kenna, an 18-year-old man in St Patrick’s Institution, a former borstal for offenders up to 21-years-old, attempted suicide by self-immolation on 14 April; later the same week Leo Byrne, a 30-year-old man on remand in the Bridewell Garda Station in Dublin, hanged himself; less than two weeks later on 28 April, John Donnellan, a 50-year-old man in Mountjoy Prison, hanged himself; and a little over two weeks after that, on 15 May, John McCarthy, another 18-year-old man in St Patrick’s Institution, hanged himself in his cell.^117^ In each case the PRO demanded a full public inquiry into conditions in the prison system, as they had at the inquest into the death of Joseph Kavanagh. It is indicative of the changing public attitude towards prison conditions that while in 1973 the newspapers relegated this demand to the end of the short article, concluding with the coroner’s dismissal of the call: ‘a public enquiry was held here today’, by 1975, after the inquest into the death of John Donnellan, it had become the headline of a large news story: ‘Inquest call for public inquiry into the prison system.’^118^ Similarly, while the early PRO protests did not attract much political attention, by May 1975, after the death of Kevin Kenna, Charlie Haughey, the former Minister for Justice then the opposition spokesman for health and social welfare, announced in a Dáil debate that ‘there is something radically wrong with the situation which prevails in our prisons in regard to the provision of psychiatric services and perhaps health services generally’, for which he was criticised for jumping on the ‘bandwagon’ of popular critiques of the prison system propagated by the PRO.^119^ In June, a month later, the PRO’s talking points were repeated almost word-for-word in questions asked in the Dáil by Dr William Loughnane and other Fianna Fáil TDs echoed the PRO’s call for a full inquiry and questioned the psychiatric provision in prisons.^120^ Indeed, Loughnane’s approach to the topic was so recognisably that of the PRO that the Minister for Justice, Cooney, repeatedly asked where the deputy had got his information, concluding dismissively:

I do not know the source of Dr Loughnane’s misinformation but there are people interested in this case – I am not saying the Dr Loughnane is one of them but he may be duped by them – whose *bona fides* towards our prisons and our prison system must be seriously in doubt. If they have been the source of information[…] then I am not surprised that the information is inaccurate and slanderous of the medical authorities in our prisons.^121^

However, for all of the Ministers’ attempts to delegitimise the PRO’s voice on penal reform, the Organisation was, by that stage, an accepted feature of the inquests into prisoners’ deaths and their statements were often uncritically repeated by the press.

The cluster of incidents of self-harm and suicide in 1975 sparked a new wave of activism. A little over two weeks after the inquest into the death of John Donnellan, the PRO held a press conference with Nora Donnellan, his wife; Christina Kenna, the mother of Kevin Kenna who set fire to himself in St Patrick’s; and Bernadette Crawley, the mother of Karl Crawley.^122^ At the press conference each of the women told the stories of their loved ones and, supported by the PRO, explained how the prison system had failed them. Crawley, in particular, soon became an important focus of PRO campaign. At the time of the press conference Karl Crawley, a 23-year-old, had spent six years in and out of St Patrick’s and Mountjoy, during which time he had attempted suicide 17 times.^123^ The PRO used the stories of these three men and the emotional weight of their relatives to argue that prisons needed a dedicated psychiatric service.

Crawley’s story gained further attention the same month when he appeared in court for assaulting a Garda while on remand. As noted earlier, the PRO had picketed the court and put leaflets on the jury’s benches. The leaflets argued that Crawley needed care in the CMH but, because they mentioned that he was currently incarcerated, they were deemed to be prejudicial. Although Crawley’s case was not one that would usually have been widely reported, the attention from the PRO, their trial, and appeal, lifted Crawley into the public view. Newspapers reported his acquittal on grounds of self-defence (his cellmate testified that the Garda had assaulted Crawley when he asked for water).^124^ They reported on Crawley’s transfer to Meath Hospital after swallowing five knife handles, two five-inch pieces of metal, and a pipe cleaner. They also reported on the prison officers’ five-day delay in believing him.^125^ The following year, when Crawley with the aid of the PRO took a High Court case to be released from Mountjoy it received widespread press coverage. During the case it emerged that during his most recent sentence Crawley had been moved to the CMH 11 times and that, while in Mountjoy, he was kept in solitary confinement in the basement and handcuffed every time he left his cell. A psychiatrist gave evidence that there were no appropriate facilities to treat him in the CMH, Mountjoy, or, indeed, anywhere in the state. Acting on Crawley’s behalf, Patrick MacEntee, a barrister with an international reputation for defending civil and political rights, argued that Crawley’s treatment in Mountjoy amounted to torture and that the state could not continue it.^126^ The court found against Crawley and the judgment argued that Crawley’s treatment, although not ideal, was an attempt to keep him from further injuring himself.

The press’ coverage demonstrated how much newspapers’ approach to prisoners had changed since the beginning of the prisoners’ rights movement. They were remarkably sympathetic to Crawley’s case, reporting that the President of the court remarked that he had to hand down this judgment ‘no matter where his sympathies might lie’, and suggesting that the state may have a duty to build a specialised facility to care for people like Crawley.^127^ The newspapers themselves echoed this tone, noting how the judgment had been given in spite of ‘the harshness of the privations which he [Crawley] had undergone, and, to a lesser extent, continued to suffer’.^128^ They spent significantly longer on Crawley’s case than that of the state, repeatedly suggesting that while the judgment was final it was not ideal and that reform was need in the psychiatric care of prisoners.^129^ By using the names and stories of specific prisoners, the PRO brought a new perspective to public discussions about prisoners’ rights. Rather than an undifferentiated mass of criminals and their gaolers, the PRO created a more nuanced image. Their main focus was on creating an image of prisoners as people with normal motivations and troubles, who, whatever their crimes (and crimes were seldom, if ever, mentioned by the Organisation), deserved proper healthcare, education, and humane treatment.

In 1975 the Minister for Justice, Patrick Cooney, dismissed the prisoners’ rights campaign as an attempt to embarrass the state. It was an accusation which the PRO was happy to confirm:

This embarrassment has always been there; only stringent secrecy has prevented its reaching the media and public until now… if the PRO has been instrumental in bringing to light the inhumanities in our prisons then we are happy to be accused of embarrassing the State.^130^

The prisoners’ rights movement in Ireland emerged as part of a complex network of circumstances, which were at once both local and international. The deterioration of prison conditions coincided with a rapid increase in the prison population followed by a rapid increase in the length of prisoners’ sentences. These institutional conditions coincided with the re-emergence of armed struggle on the Island of Ireland, an increasingly internationally-networked civil rights movements, the growing international weight of human rights discourse, and a wave of prisoner activism that reached from America to Australia. In this complex situation the prisoners’ rights movement used every tactic available to them. The institutionally powerless and marginalised prisoners banded together to form the PU, at first using collective action, and later using increasingly desperate and individual forms of self-harm as protest. Later the PRO marched with placards outside prisons and held increasingly well received press conferences. Gradually they pushed their way into coroners’ inquests, trials, and the pages of national newspapers. At every step they attempted to make their voice louder and more legitimate through the voices of their spokespeople, the language that they used, and the tactics they employed. As they gathered social power, they were able to make have an institutional impact at coroners’ inquests and to influence Dáil debates. Ultimately, this article has argued, they were about to use their increasingly legitimate public voice to reframe the debate about prisons, highlighting prisoners’ experiences and the human impact of the prison system.

## Footnotes

1 Anon, ‘Prisoner Swallows Metal’, *Irish Press* (24 September 1975), 1.

2 Gene Kerrigan, ‘Special Feature: A Necessary Respect – The Small Legend of Karl Crawley’, *Magill* (31 May 1982), np. While incidents were rarely reported on in the Irish media, Chris Millard notes that there was a well-established post-war psychological literature on prisoners’ self-harm as a ‘response to the confined space, or to perceived injustices’. Chris Millard, *A History of Self-Harm in Britain: A Genealogy of Cutting and Overdosing* (Houndmills 2015), 168. Catherine Cox and Hilary Marland, ‘Broken Minds and Beaten Bodies: Cultures of Harm and the Management of Mental Illness in Mid- to Late Nineteenth-Century English and Irish Prisons’, *Social History of Medicine*, 31, 4 (2018), 692; Sarah Chaney, *Psyche on the Skin: A History of Self-Harm* (Islington 2017).

3 Unfortunately, I have only been able to find an incomplete set of the *Jail Journal* at the National Library of Ireland.

4 For instance, Anon, ‘Daingean Reformatory’, *Jail Journal,* 1, 1 (1973), 6–7; Anon, ‘The Young Offender’, *Jail Journal,* 1, 5 (1974), 9; Anon, ‘A Prisoner Remembers’, *Jail Journal,* 1, 7 (1975), 3. Compared to Joe Costello, ‘Public Enquiry Petition Presented’, *Jail Journal,* 2, 3 (1977), 8–9; Anon, ‘Legal Aid’, *Jail Journal,* 2, 4 (1977), 2–3; Anon, ‘Life in the City Centre’, *Jail Journal,* 2, 6 (1978), 10–1.

5 Mick Ryan, *The Acceptable Pressure Group: Inequality in the Penal Lobby a Case Study of the Howard League and RAP* (Farborough 1978), 151–2; Mick Ryan, *Penal Policy and Political Culture in England and* Wales (Winchester 2003), 19–20; Mick Ryan, *The Politics of Penal Reform* (Harlow 1983), 78, 113.

6 Anon, ‘Prisoners’ Committee’, *Irish Independent* (25 November 1977), 10.

7 William Murphy, *Political Imprisonment and the Irish, 1912–1921* (Oxford 2014), 1–2.

8 Ian Miller, *A History of Force Feeding: Hunger Strikes, Prisons and Medical Ethics, 1909–74* (Basingstoke 2016), 2; Vicky Conway and Jennifer Schweppe, ‘What is a Miscarriage of Justice? The Irish Answer to an International Problem,’ *Dublin University Law Journal*, 35 (2012), 1–33, 18–9.

9 See for example: Seán McConville, *Irish Political Prisoners, 1848–1922: Theatres of War* (Routledge 2003); David Beresford, *Ten Dead Men: The Story of the 1981 Irish Hunger Strike* (London 1987); George Sweeney, ‘Irish Hunger Strikes and the Cult of Self-Sacrifice’, *Journal of Contemporary History*, xxviii (1993), 421–37; Seán O’Mahony, *Frongoch: University of Revolution* (Dublin 1987); Padraic Kenney, ‘“I Felt a Kind of Pleasure in Seeing Them Treat us Brutally”: The Emergence of the Political Prisoner, 1865–1910’, *Comparative Studies in Society and History,* 54, 4 (2012), 863–89. There have also been several biographical reflections on protests in prison, for instance: Margaret Ward, *Hanna Sheehy Skeffington: A Life* (Dublin 1997); and David Fitzpatrick, *Harry Boland’s Irish Revolution* (Cork 2003).

10 Ian Miller, *A History of Force Feeding: Hunger Strikes, Prisons and Medical Ethics, 1909–1974* (Basingstoke 2016), 204–5; Mary Rogan, *Prison Policy in Ireland: Politics, Penal-Welfarism and Political Imprisonment*, (London 2011), 142–7; Cormac Behan, *Citizen Convicts: Prisoners, Politics and the Vote* (Manchester 2014), 76–8; Shane Kilcommins, Ian O’Donnell, Eoin O’Sullivan, and Barry Vaughan, *Crime, Punishment and the Search for Order in Ireland* (Dublin 2004), 7; Diarmaid Ferriter, *Ambiguous Republic: Ireland in the 1970s*, (London 2012), 358; Kilcommins, O’Donnell, O’Sullivan, and Vaughan, *Crime, Punishment and the Search for Order in Ireland*, 71.

11 Rogan, *Prison Policy in Ireland*, 145.

12 In a letter to a former member of the Sean MacBride Commission, which the PRO established as a way of pressuring the government into holding a full enquiry, Whitaker remarked of their report: ‘Indeed when I read what you have written […] I wonder why another body was set up.’ T. K. Whitaker, ‘Letter from T. K. Whitaker to Una O’Higgins O’Malley’, 26 March 1984, University College Dublin Archives P175/133.

13 Cormac Behan, ‘“We Are All Convicted Criminals”? Prisoners, Protest and Penal Politics in the Republic of Ireland’, *Journal of Social History*, 51, 3 (Fall 2018), 502.

14 Behan characterises the PU as a ‘flag of convenience’, which would explain the periodic re-emergence of the PU, for instance during the 1977 hunger strike in the Curragh (Ibid, 516; Anon, ‘Hunger Strike’, *Jail Journal*, 1, 12 (April 1977), 2.). However, as we will see, the first incarnation of the PU in 1973 modelled itself on the formal organisation of a trade union suggesting that, at this time, it was more than a flag of convenience. When discussing the PU, this article focuses on its initial phase when it was at its most organised, powerful, and coherent (January-November 1973).

15 At the beginning of this study in 1972 the average daily population of women in prison was 26 (compared to 1,009 men), and by 1976 it had only risen to 28 (compared to 1,021 men). Department of Justice, *Annual Report on Prisons for 1972* (TAOIS/2005/7/244, National Archives of Ireland), 30; Greg Heylin, *Evaluating Prisons, Prisoners and Others* (Dublin 2001), 116.

16 Department of Justice, Annual Report on Prisons for 1972, 30.

17 Anon, ‘The Women’s Prison: The Facts of the Horror’, *Jail Journal*, 1, 2 (1973), 6.

18 For instance: Anon, ‘The Good Life’, *Jail Journal,* 1, 1 (1973), 12; Anon, ‘T. I. C.’, *Jail Journal,* 1, 1 (1973), 13; Anon, ‘An Alcoholic’s Experience’, *Jail Journal,* 1, 3 (1974), 14–16.

19 Ian O’Donnell, Eoin O’Sullivan, and Deirdre Healy (eds), *Crime and Punishment in Ireland 1922 to 2003: A Statistical Sourcebook* (Dublin 2005), 145–9.

20 Kilcommins, O’Donnell, O’Sullivan, and Vaughan, *Crime, Punishment and the Search for Order in Ireland*, 68–70.

21 Rogan, *Prison Policy in Ireland*, 107.

22 Patrick Cooney, ‘Committee on Finance – Vote 20: Office of the Minister for Justice’, *Dáil Debates*, 12 February 1974.

23 O’Donnell, O’Sullivan, and Healy (eds), *Crime and Punishment in Ireland 1922 to 2003*, 151.

24 Ibid, 145–9, 16–7, 32–3, 48–9.

25 Anon, ‘The Prison Visiting Committees,’ *Jail Journal*, 1, 12 (1977), 6–9. See p. 7.

26 Department of Justice, *Rules for the Government of Prisons* (Dublin 1947), Arts 54.1 and 54.2. There had also been schools in prisons during the nineteenth century, although they were unevenly spread between the various gaols Beverly A. Smith, ‘The Female Prisoner in Ireland, 1855–1878,’ *Federal Probation*, 54, 4 (1990), 69–81. See p. 61.

27 Prison Study Group, *An Examination of the Irish Penal System* (Dublin 1972), 49, 56, 61.

28 Prison Study Group, *An Examination of the Irish Penal System*, 44–5, 58. The rural nature of the work in Portlaoise was particularly given the urban background of the majority of the prisoners there.

29 While there were no studies of the mental health of prisoners in Ireland, these claims, put forward by the PRO in 1976, are based on a British Home Office report. Anon, ‘Prison and the Mentally Ill’, *Jail Journal*, 1, 9 (1976), 12.

30 Prison Study Group, *An Examination of the Irish Penal System*, 51–2.

31 Ibid, 50–1, 57, 62.

32 RTÉ, *Central Mental Hospital Dundrum*, 1971, RTÉ Archive; Prison Study Group, *An Examination of the Irish Penal System*, 54–5.

33 Prison Study Group, *An Examination of the Irish Penal System*, 54–5.

34 Nicholas Duvall, ‘“From Defensive Paranoia to… Openness to Outside Scrutiny”: Prison Medical Officers in England and Wales, 1979–86’, *Medical History*, 62, 1 (2018), 112–31. See p. 120.

35 Lee Bernstein, ‘The Age of Jackson: George Jackson and the Culture of American Prisons in the 1970s’, *The Journal of American* Culture, 30, 3 (2007), 310–323. See pp. 312–7.

36 Anon, ‘Riot Jail Deaths Riddle,’ *Irish Independent* (September 15, 1971) 28; Anon, ‘Brutality Alleged After Jail Uprising,’ *Evening Herald* (October 1, 1971), 11.

37 Mike Fitzgerald, *Prisoners in Revolt: The Origin and Development of Preservation of the Rights of Prisoners (PROP), The British Prisoners Union*, PhD thesis, University of Leicester (1976), 1.

38 David Scott, ‘Resistance as Reform: Direct Action Through Prisoner Movements, Legal Activism and the Radical Penal Lobby,’ *Criminal Justice Matters* 77, 1 (2009), 20–1. See p. 20.

39 Michael Welch, ‘Pastoral Power as Penal Resistance: Foucault and the Groupe d’Information sur les Prisons’, *Punishment & Society*, 12, 1 (2009), 47–63. See p. 58.

40 Cecile Brich, ‘The Groupe d’Information sur les Prisons: The Voice of Prisoners? Or Foucault’s?’, *Foucault Studies*, 5 (2008), , 26–47. See p. 26.

41 Prisoners’ Action Group, ‘Bathurst Gaol and the Royal Commission into Prisons: A Summary by the Prisoners’ Action Group,’ *Alternative Criminology Journal* 2, 3 (1978), 1–42. See pp. 16–7.

42 George Zdenkowski and David Brown, *The Prison Struggle: Changing Australia’s Penal System* (Harmondsworth 1982), 80, 99.

43 Rogan, *Prison Policy in Ireland,* 147.

44 Kilcommins, O’Donnell, O’Sullivan, and Vaughan, *Crime, Punishment and the Search for Order in Ireland*, 235–7. Minor reforms were carried out during the decade. The prison diet was varied, new industrial training programmes were introduced in Mountjoy, and a work day-release programme was developed. However, these were extremely limited and had little structural impact. Rogan, *Prison Policy in Ireland*, 133.

45 Anon, ‘Prison Back to Normal in Portlaoise’, *The Nationalist and Leinster Times* (17 November 1972), 1. The estimation that this is almost half the prison’s population is based on the average daily prison population of 178.97, although this was not a stable number fluctuating between a low point of 147 and a high of 208. O’Donnell, O’Sullivan, and Healy (eds), *Crime and Punishment in Ireland*, 151.

46 Anon, ‘Prison Back to Normal in Portlaoise’.

47 Simon O’Donnell, ‘Portlaoise Prisoners’ Union’, *Irish Press* (24 May 1973), 10; Anon, ‘Committee on Prison Reform Dossier for Minister’, *Irish Press* (19 May 1973), 3.

48 Anon, ‘Prison Back to Normal in Portlaoise’, *The Nationalist and Leinster* Times (17 November 1972), 1.

49 O’Donnell, ‘Portlaoise Prisoners’ Union’; Anon, ‘Committee on Prison Reform Dossier for Minister’.

50 Anon, ‘Prisoners on Diet’, *The Nationalist* (24 November 1972), 19.

51 Ibid.

52 William Murphy, *Political Imprisonment and the Irish, 1912–1921* (Oxford 2014), 12, 188.

53 Ian Miller, *A History of Force Feeding: Hunger Strikes, Prisons and Medical Ethics, 1909-1974* (Basingstoke 2016), 157, 166–7.

54 Interestingly, out of the 849 strikes in which the motivation was recorded by prison authorities only 40 (4.7 per cent) were politically motivated. Ibid, 154.

55 Natcha Filippi, ‘Women’s Protests: Gender, Imprisonment and Resistance in South Africa (Pollsmoor Prison, 1970s–90s)’, *Review of Political Economy*, 43, 149 (2016), 436–50. See p. 444; Marcello Hoffman, ‘Foucault and the “Lesson” of the Prisoner Support Movement’, *New Political Science*, 34, 1 (2012), 21–36. See pp. 24–5.

56 See for example: Bea Brockman, ‘Food Refusal in Prisoners: A Communication or a Method of Self-Killing? The Role of the Psychiatrist and Resulting Ethical Challenges’, *Journal of Medical Ethics*, 25, 6 (1999), 451–6; Art O’Connor and Eric Johnson-Sabine, ‘Hunger Strikers’,* Medicine, Science, and the Law*, 28, 1 (1988), 62–4; Mikki Gorsuch, ‘Disturbed Female Offenders: Helping the “Untreatable”’, *Journal of Forensic Psychiatry*, 10, 1 (2008), 98–118.

57 Anon, ‘Garda Alert on Protests in Two Jails’, *Irish Independent* (8 January 1973), 1.

58 O’Donnell, ‘Portlaoise Prisoners’ Union’.

59 Irish Press Reporter, ‘Prisoners are Being Illtreated SF Charge Government’, *Irish Press* (2 February 1973), 4.

60 Anon, ‘Prisoners Threaten to Strike’, *Irish Press* (21 February 1973), 5.

61 Anon, ‘Prison Protest’, *Nationalist and Leinster Times* (2 March 1973), 1.

62 Denis Dennehy, ‘Portlaoise Prisoners’ Union’, *Irish Press* (30 May 1973), 10.

63 Marguerite Helmers, ‘Handwritten Rebellion: Autograph Albums of Irish Republican Prisoners in Frognach’, *New Hibernia Review*, 22, 3 (2018), 20–38. See p. 36.

64 George Sweeney, ‘Irish Hunger Strikes and the Cult of Self-Sacrifice’, *Journal of Contemporary History*, xxviii (1993), 421–37; Kevin Grant, ‘British Suffragettes and the Russian Method of Hunger Strike’, *Comparative Studies in Society and History*, 53, 1 (2011), 113–43, See p. 114.

65 Anon, ‘Garda Alert on Protests in Two Prisons’.

66 Department of Justice, *Memorandum for the Government: Proposed Bill to amend the Prisons Act, 1972*, Unpublished memo (April, 1974), National Archives of Ireland Jus/2008/156/2 1/3.

67 O’Donnell, ‘Portlaoise Prisoners’ Union’.

68 Conor McAnally, ‘Prisoners in Roof Sit-Down’, *Irish Independent* (15 September 1973), 1.

69 Maire Bates, ‘Portlaoise Escape’, *Irish Press* (29 August 1974), 8.

70 Jim Farrelly, ‘Blackmail Complaints by Prison Officers’, *Evening Herald* (18 August 1973), 1.

71 Alfie Jones, ‘Prison Conditions’, *Irish Press* (19 June 1974), 10.

72 Anon, ‘Prison Officers’, *Jail Journal* 1, 6 (1974), 6–7.

73 Anon, Cartoon, *Jail Journal* 1, 7 (1975), 9.

74 Anon, ‘Prisoners’ Demands “Rejected”’, *Irish Press* (3 February 1973), 6.

75 Ibid.

76 O’Donnell, ‘Portlaoise Prisoners’ Union’.

77 John Kearns, ‘Prisoners’ rights’, *Irish Press* (7 July 1973), 8; Anon, ‘Ex-Prisoner Attacks “Degrading” life behind bars’, *Evening Herald* (18 May 1973), 6.

78 Prisoner Rights Organisation, ‘Demands of the Portlaoise Prisoners’ Union (Smuggled out on 8/5/’73)’, *Jail Journal*, 1, 5 (1974), 2.

79 John Kearns, ‘Committee for Prison Reform’, *Evening Herald* (9 July 1973), 6; Kearns, ‘Prisoners’ Rights’; Anon, ‘Prisons Reform Urged by Picket at Jail’, *Irish Independent* (30 July 1973), 7; Anon, ‘Probe Into “Sale of Jail Goods”’, *Irish Press.* (2 August 1973), 4; Noel T. Lemass and John P. Wilson, Dáil Éireann debate (17 July 1973).

80 Irish Independent Staff Reporter, ‘Prison Protest’, *Irish Independent* (24 May 1973), 1.

81 Anon, ‘Cooney Hits as Prisoners’ Union’, *Cork Examiner* (26 May 1973), 1.

82 Anon, ‘Letter from Portlaoise – Hoping for a New Deal’, *Evening Herald* (8 September 1973), 6.

83 The other two prisoners were Dick Power and Tom Savage. Anon, ‘Jail Roof Marathon: Portlaoise Protest Enters Second Day’, *Irish Press* (15 September 1973), 1.

84 Anon, ‘Jail Roof Marathon: Portlaoise Protest Enters Second Day’.

85 Anon, ‘Prisoners’ Roof Protest’, *Irish Independent* (15 September 1973), 24.

86 Anon, ‘Jail Roof Marathon’.

87 Conal Gibbons, ‘Portlaoise Prison Protest’, *Irish Independent* (25 September 1973), 8.

88 Anon, ‘“Bread and Water Diet” Allegation’, *Munster Express* (21 September 1973), 5.

89 Department of Justice, *Memorandum for the Government*.

90 Anon, ‘Knives Used in Jail’, *Irish Independent* (8 October 1973), 3.

91 Ibid.

92 Anon, ‘Prisoners to Stage Peaceful Protest’.

93 Anon, ‘Prisoners Leaders Sent to Curragh’, *Irish Independent* (14 November 1973), 12; Anon, ‘Prison Union Leaders Moved’, *Irish Independent* (12 November 1973), 12.

94 There may have been a brief re-emergence of the PU in 1977, but it did not gather much attention, featuring in only one article in the Jail Journal and briefly alluded to in an article in the Irish Press. Anon, ‘Hunger strike,’ *Jail Journal*, 1, 12 (1977), 2; Tom Fallon, ‘Watchdogs in the Public Interest,’ *The Irish Press* (26 December 1977), 6.

95 Pat Holmes, ‘A Special Report From’, *Irish Press* (22 November 1973), 6.

96 Anon, ‘Multi-Million Prisons Plan’, *Cork Examiner* (22 November 1973), 9.

97 Anon, ‘Ex-Prisoner Says Irish Jails “Deplorable”’.* Cork Examiner* (19 May, 1973), 11; Civis, ‘Counterpoint’* Nationalist and Leinster Times* (8 June 1973), 23. Bourke was active with the PRO in 1973 and 1974 but then does not appear in their literature after this. He may have returned to his career as a house painter, and in 1976 one Tom Bourke, of the Dublin suburb or Raheny, advertised reasonable rates for ‘painting, papering, and graining’. Anon, ‘Useful Services,’ *Evening Herald* (18 March 1976), 14.

98 Anon, ‘New Group is Seeking Prison Reform’, *Nationalist and Leinster Times* (25 May 1973), 9.

99 Kearns, ‘Prisoners’ Rights’; Kearns, ‘Committee for Prison Reform’.

100 Prisoner Rights Organisation, ‘Introduction’, *Jail Journal*, 1, 1 (1974), 2–3.

101 Anon, ‘Prison Reform Urged by Picket at Jail’, *Irish Independent* (30 July 1973), 7.

102 Anon, ‘Picket at Ministerial Function’, *Irish Examiner* (29 August 1973), 7.

103 Anon, ‘Protest Over Prison Conditions’, *Irish Press* (26 September 1973), 4.

104 Anon, ‘Prisoners to Stage Peaceful Protest’, *Evening Herald* (26 October 1973), 6.

105 Declan White, ‘Prison Trouble After Portlaoise Breakout?’, *Sunday Independent* (25 August 1974), 3; Irish Press Reporter, ‘Hunger Strike at Curragh’, *Irish Press* (24 October 1975), 4.

106 Anon, ‘Picketing Ban on Eight’, *Irish Examiner* (10 May 1975), 12.

107 Anon, ‘Civil Servants Gets Picket Outlawed’, *Irish Press* (10 May 1975), 5.

108 Wladek Gaj, Private correspondence.

109 Anon, ‘Court Picket Win Appeal, Sentences Quashed’, *Irish Press* (12 November 1975), 5.

110 Anon, ‘Civil Servants Gets Picket Outlawed’.

111 Suicides were not usually reported in Visiting Committee or Annual Prisons Reports and there are no official sources for statistics about self-harm in prison in this period. It is, however, worth noting that newspapers did report on suicides of prisoners in the Central Mental Hospital during this period, so it may be fair to suggest that they would have reported on suicides in prisons as well and, therefore, to infer that there were no such deaths. However, this lack of evidence is in no way conclusive.

112 O’Donnell, ‘Portlaoise Prisoners’ Union’.

113 Anon, ‘Death in Prison’, *Jail Journal*, 1, 1 (1973), 14–5.

114 Anon, ‘Man Found Dead in Cell Inquest to be Held’, *Irish Independent* (6 August 1973), 11.

115 Ibid.

116 Anon, ‘Inquest Urges Jail “Medical”’.

117 Anon, ‘Burned Convict. P.R.O. Demand Inquiry’, *Irish Independent* (21 April 1975), 1.; Anon, ‘No Medical Supervision Blamed for Cell Deaths’, *Sunday Independent* (4 May 1975), 15; Anon, ‘Inquest Call for Public Inquiry into the Prison System’, *Irish Independent* (17 May 1975), 3; Anon, ‘Accident Verdict on Cell Death’, *Irish Press* (20 June 1975), 3.

118 Anon, ‘Inquest Urges Jail “Medical”’, *Irish Press* (16 October 1973), 3; Anon, ‘Inquest Call for Public Inquiry into the Prison System’.

119 Dáil Éireann debate, ‘Vote 49: Health (resumed)’, 21 May 1975; Dáil Éireann debate, ‘Vote 49: Health (resumed)’, 23 May 1975.

120 Dáil Éireann debate, ‘Questions (Death of a Prisoner)’, 18 June 1975.

121 Dáil Éireann debate, ‘Adjournment Debate (Death of a Prisoner)’, 18 June 1975.

122 Anon, ‘Mothers Fear for Sons’ Lives, Jail Needs Psychiatrists’, *Irish Press* (4 June 1975), 7.

123 Anon, ‘Adjournment in Assault Case’, *Evening Herald* (20 June 1975), 4.

124 Anon, ‘Court Picket Win Appeal, Sentences Quashed’.

125 Anon, ‘Prisoner Swallows Metal’.

126 In the same year as Crawley’s trial McEntee went to pre-independence Namibia as an observer for the International Commission of Jurists where he provided international oversight of a trial of political activists. Source?

127 Anon, ‘Man’s Plea for Release Rejected’, *Irish Independent* (14 April 1976), 11.

128 Anon, ‘Judge Rules on Special Care’, *Irish Press* (14 April 1976), 6.

129 Anon, ‘Man’s Plea for Release Rejected’; Anon, ‘Prisoner Refused Release Order’*, Cork Examiner* (14 April 1976), 7; Anon, ‘Judge Rules on Special Care’.

130 Anon, ‘Prison Group Challenges Minister’, *Irish Press* (19 May 1975), 5; Anon, ‘Prisons Exposed by Suicides’, *Irish Press* (26 May 1975), 5.

